# Bioresource Potential and Safety Evaluations of Thai *Zea mays* L. Husk Waste Extracts

**DOI:** 10.3390/foods15111906

**Published:** 2026-05-28

**Authors:** Mathukorn Sainakham, Wantida Chaiyana, Kanokwan Kiattisin, Suvimol Somwongin, Worrapan Poomanee, Vanuchawan Wisuitiprot

**Affiliations:** Department of Pharmaceutical Sciences, Faculty of Pharmacy, Chiang Mai University, Chiang Mai 50200, Thailand; mathukorn.s@cmu.ac.th (M.S.); wantida.chaiyana@cmu.ac.th (W.C.); kanokwan.k@cmu.ac.th (K.K.); suvimol.s@cmu.ac.th (S.S.); worrapan.p@cmu.ac.th (W.P.)

**Keywords:** corn husk extracts, bioactivity, HET-CAM, green extraction, value-added upcycling

## Abstract

Corn husk, a prevalent agricultural byproduct, remains an underutilized source of bioactive compounds. This study investigated the influence of extraction solvents (water, 50% ethanol, and 95% ethanol) and techniques (maceration, reflux, ultrasound-assisted extraction) on the phytochemical profiles and biological activities of corn husk. The results revealed that water extraction produced the highest total phenolic content, whereas 95% ethanol extraction yielded the greatest total flavonoid content and the most potent antioxidant activity in DPPH testing. In contrast, ultrasound-assisted water extraction exhibited the most potent nitric oxide inhibition (94.62 ± 2.13%) and tyrosinase inhibition (IC_50_ = 7.54 ± 0.27 mg/mL), indicating anti-inflammatory and skin-lightening potential. This extract showed anti-collagenase activity (91.49 ± 4.01%), outperforming ascorbic acid (29.79 ± 1.00%) and EGCG (82.27 ± 1.00%), though its anti-hyaluronidase activity was limited. Cytotoxicity testing revealed cytotoxicity at 10 mg/mL, while the HET-CAM assay confirmed non-irritation at the same level. These findings underscore that ultrasound-assisted water extraction is a safe and successful technique for obtaining bioactive-rich extracts. This study supports the transition of corn husk from agricultural waste to a high-value, safe, and multifunctional natural ingredient for the cosmetic, pharmaceutical, and functional food industries.

## 1. Introduction

Global warming is fundamentally a catastrophic thermal imbalance resulting from an unprecedented concentration of greenhouse gases. This challenge fundamentally stems from carbon-intensive emissions resulting from energy production, industrial processes, and widespread deforestation. Practices like the burning of agricultural waste intensify damage, pushing the world climate to a dangerous and potentially irreversible tipping point [1,2]. Agriculture creates solid waste that makes up 10% to 60% of the resources necessary to process it. There are also times when the waste products are worth more than the main parts. Most of these by-products are thrown away. Some of these by-products are the epidermis, seeds, stalks, leaves, effluents, and pulp that cannot be consumed [3]. However, the rise of the Bio-Circular-Green (BCG) economy has shifted the perspective on agricultural waste, reclassifying it as a vital source of high-value natural molecules. There is a burgeoning industrial demand for bio-based alternatives to synthetic chemicals in the pharmaceutical, nutraceutical, and cosmeceutical sectors [4]. Corn (*Zea mays* L.) is a fundamental component of world agriculture; nonetheless, its husk is a notably underexploited lignocellulosic resource. In Thailand, one of the region’s primary corn producers, massive quantities of corn husk are discarded annually due to limited awareness of their sophisticated phytochemical profiles. The recent literature suggests that corn husk is not merely fiber; it is a dense matrix of lipophilic carotenoids such as lutein and zeaxanthin extracted by ethanol at elevated temperatures. It may inspire health product development due to having the highest antioxidant activity, while flavonoid-rich extracts were also effectively extracted from corn husks by enzyme-assisted extraction [5]. Bound phenolic acid, ferulic acid, also isolated from corn husk, could lead to the apoptosis of MDA-MB-231 cells; their cell viability was increased by inhibiting G2/M phase of the cell cycle concomitantly with increasing levels of cellular reactive oxygen species [6]. In addition, reducing sugar was found in corn husk methanolic extract; the extract also showed that antidiabetic activities were determined based on α-amylase inhibitory activity, exhibiting an IC50 = 2.77 mg/mL [7]. Although a few reports could indicate the specific chemical compound, the corn husk extract also presented the beneficial bioactivities; a study revealed that corn husk extract could suppress inflammatory processes by inhibiting the expression of iNOS gene and NF-kB and AP-1 signaling in LPS-induced RAW264.7 cells [8]. These constituents are documented to exhibit beneficial activities, positioning corn husk as a premium feedstock for functional food and cosmetic product development. Despite this potential, the biological efficacy of these compounds is strictly dependent on their structural integrity post-extraction. The conversion from raw biomass to a bioactive ingredient is often obstructed by inadequate recovery methods. The influence of different extraction parameters on the optimization of these specific bioactivities remains largely unexplored; there is a critical need for defined protocols. Consequently, the present study aims to evaluate and compare the impact of different extraction techniques on the phytochemical profiles and functional bioactivities of corn husk, providing a technical framework for its industrial valorization.

## 2. Materials and Methods

### 2.1. Materials

Dried corn husk samples were purchased from a farmer in Lampang Province, Thailand. DPPH and Folin–Ciocalteu were obtained from Fluka (Buchs, Switzerland) and Merck (Darmstadt, Germany), respectively. Analytical chemicals such as gallic acid, quercetin, and MTT were supplied by Sigma (St. Louis, MO, USA). For the cell culture study, FBS, BSA, DMEM, and penicillin–streptomycin were purchased from Invitrogen (Grand Island, NY, USA). Solvents and additional reagents, such as ethanol and DMSO, were supplied by Labscan Asia Co., Ltd. (Bangkok, Thailand). Unless otherwise stated, every chemical used in this study was of analytical grade. The human dermal fibroblasts (ATCC CRL-2932) were obtained from the American Type Culture Collection (ATCC, Manassas, VA, USA).

### 2.2. Extraction of Botanical Substances

**Figure 1:**
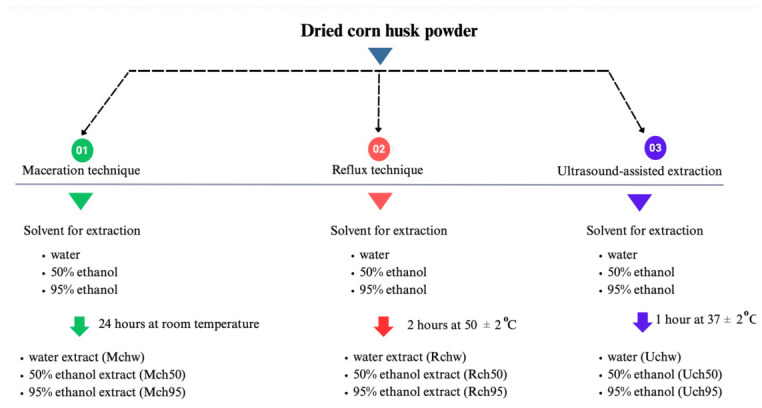
The flowchart illustrates the extraction procedure of corn husk extract and abbreviations. Mchw = maceration with water; Uchw = ultrasound-assisted extraction with water; Rchw = reflux extraction with water; Mch50 = maceration with 50% ethanol; Uch50 = ultrasound-assisted extraction with 50% ethanol; Rch50 = reflux extraction with 50% ethanol; Mch95 = maceration with 95% ethanol; Uch95 = ultrasound-assisted extraction with 95% ethanol; and Rch95 = reflux extraction with 95% ethanol.

An amount of dry corn husk powder was obtained utilizing 95% ethanol, 50% ethanol, and water. The extraction conditions and their abbreviations are shown in Figure 1. Samples were extracted through maceration in solvents for 24 h at ambient temperature using a non-heating approach, while samples with distilled water were heated by an electric hot plate for 2 h. The samples from 95% and 50% ethanol were refluxed for 2 h at 50 ± 2 °C. Ultrasound-assisted extraction using an ultrasonic cleaner (Elmasonic S100 H, JP-50 kHz, 550 W; Elma, Germany) was performed on the samples utilizing these solvents for one hour at 37 ± 2 °C. Following filtration through Whatman No. 1 paper, a rotary vacuum evaporator (M.A-3S, Eyela, Japan) was employed to evaporate the ethanolic extract’s solvent, whereas lyophilization (Christ Beta 2-8 LD plus, Osterode am Harz, Germany) was employed to concentrate deionized water extracts. All concentrated extracts were subsequently preserved at 4 °C for further analysis.

### 2.3. Investigation of Total Phenolic Content (TPC) and Total Flavonoid Content (TFC)

The phenolic content was quantified via the Folin–Ciocalteu method and expressed as milligrams of gallic acid equivalents (GAE) per gram of extract. The strategy outlined in the published article was employed with minor adjustments [9]. In summary, 20 µL of corn husk extract at a concentration of 1 mg/mL was combined with 180 µL of 1:10 diluted Folin–Ciocalteu reagent and permitted to incubate at room temperature for 4 min. Subsequently, 80 µL of sodium carbonate solution (75 mg/mL) was included and subsequently held at ambient temperature for an additional 2 h. Absorbance was quantified at 750 nm utilizing a microplate reader. Gallic acid served as a standard, and the total phenolic content was quantified as mg/g of gallic acid equivalents (GAE). The assessment of total flavonoid content with the aluminum chloride method was performed as previously described by [10]. Twenty microliters of the sample were amalgamated with 0.2 milliliters of 5% sodium nitrite and incubated at room temperature for 5 min. Subsequently, 0.2 mL of 10% aluminum chloride was added and incubated for 6 min, after which 1 mL of 1 M sodium hydroxide was administered. The solution was diluted to 2 mL using deionized water and incubated for 30 min. Absorbance was measured at 510 nm using a microplate reader. The total flavonoid content was measured using quercetin as a standard and expressed as mg QE per gram of dry weight.

### 2.4. Assay of Antioxidant Activities

As previously mentioned, the DPPH radical scavenging test and the lipid peroxidation inhibition experiment were used to investigate the extracts’ antioxidant qualities [11]. In a 96-well plate, the DPPH radical’s scavenging activity was investigated. Absorbance was measured at 517 nm after the samples were mixed with DPPH in ethanol and incubated for 30 min at 27 ± 2 °C in the dark. The sample concentration required to scavenge DPPH at 50% (IC50) was calculated. The ferric thiocyanate reaction’s activity was assessed for its ability to prevent lipid peroxidation. In a 96-well plate, the samples and reaction reagents were mixed and incubated. After one hour of incubation at 37 ± 2 °C, the complex formation resulting from ferric thiocyanate was measured at an absorbance of 490 nm. The sample concentration at which 50% inhibition of lipid peroxidation occurred was indicated by the IC50 value.

### 2.5. Assay of Anti-Tyrosinase Activity

The inhibitory impact of mushroom tyrosinase on tyrosinase was assessed utilizing L-tyrosine or L-DOPA as substrates, with minor changes as previously outlined by [12,13]. Corn husk extracts were solubilized in DMSO at a concentration of 1.0 mg/mL and subsequently diluted to various quantities using DMSO. Thirty microliters of each sample was diluted with 970 μL of 0.05 mM sodium phosphate buffer (pH 6.8) in a test tube. Subsequently, 1 mL of L-tyrosine or L-DOPA solution was incorporated, followed by the addition of 1.0 mL of mushroom tyrosinase (200 units/mL). Three milliliters of maize husk extract solution were combined and incubated for 20 min at 37 °C, after which the absorbance at 475 nm was recorded. The IC50 value indicates the concentration of the drug required to achieve 50% inhibition of tyrosinase activity.

### 2.6. Assay of Scavenging of Nitric Oxide (NO) Radical

The proportion of nitric oxide radical scavenging activity was determined as previously outlined by [11]. The reaction mixture comprised 2 mL of 10 mM sodium nitroprusside, 0.5 mL of saline phosphate buffer, and 0.5 mL of maize husk extracts, which were incubated at 25 °C for 180 min. After incubation, the test solution was combined with 50 μL of 0.1% N-(1-naphthyl) ethylenediamine dihydrochloride (NEDD) in distilled water and incubated at ambient temperature for 5 min. Subsequently, 50 μL of 1% sulfanilamide in 5% phosphoric acid was introduced, and the solution was allowed to stand for 5 min. The reaction’s absorbance was subsequently quantified with a microplate reader at 510 nm. The percentage of inhibition was determined by comparison with the negative control.

### 2.7. Assay of Anti-Aging Activity

The extracts were analyzed for their inhibitory effects on collagenase and hyaluronidase activity in a 96-well plate, using the approach described by Phumat and colleagues [14]. Collagenase from Clostridium histolyticum (ChC–EC.3.4.23.3) and bovine testis hyaluronidase were employed to react with the substrates N-[3-(2-furyl) acryloyl]-Leu-Gly-Pro-Ala (FALGPA) and hyaluronic acid, respectively. Post-incubation, the samples were assessed for absorbance via a microplate reader, and enzyme activity inhibition was determined using the following formula: % inhibition = [(A − B)/A] × 100, where A represents the reaction rate in the mixture comprising both substrate and enzyme. B represents the reaction rate in the mixture comprising the sample, enzyme, and substrate.

### 2.8. Hen’s Egg Chorioallantoic Membrane (HET-CAM) Observation

The fertilized hen eggs were incubated at 37.5 ± 0.5 °C and 62.5 ± 7.5% relative humidity. Exposure of the inner shell membrane was achieved by excising the shell at the air cell, followed by the application of normal saline onto the membrane layer in direct contact with the CAM. After the meticulous removal of the CAM, 30 µL of corn husk extract (10 mg/mL), 1% sodium lauryl sulfate, or 0.9% normal saline was administered. The capillary network of the chorioallantoic membrane was subjected to detailed stereomicroscopic examination (Olympus, Tokyo, Japan) to detect signs of irritation. Clinical symptoms, specifically hemorrhage, lysis, and coagulation, were documented throughout an observation window of 5–60 min following sample application.

The irritation score (IS) has been calculated as previously indicated after the beginning of each irritation indication was recorded [15,16].

### 2.9. Cytotoxicity Assessment via the MTT Assay

In 96-well plates, human skin fibroblasts were seeded using complete DMEM and incubated under a 5% CO_2_ atmosphere at 37 °C for 24 h. Following this equilibration period, cells were exposed to different concentrations of the samples and maintained under identical conditions for an additional 24 h. The medium was then replaced with MTT solution, followed by a 4 h incubation. The formazan crystals were then dissolved in DMSO, and the absorbance at 560 nm was measured to determine the percentage of cell viability as previously described [17].

### 2.10. Quantification of Active Constituents in the Extract by High-Performance Liquid Chromatography (HPLC) Analysis

A high-performance liquid chromatography (HPLC) system with a diode-array detector (1260 Infinity II, Agilent Technologies, Santa Clara, CA, USA) was used to measure the amounts of gallic acid and quercetin in the extracts. A 5 µm, 250 × 4.6 mm C18 column (Agilent HC-C18, Santa Clara, CA, USA) was used in this experiment. The method previously described in [18] was used to determine the parameters and mobile phase. Gallic acid and quercetin were measured at 254 nm and 370 nm wavelengths using the following gradient elution program: 0–5 min (10% B), 5–20 min (10–25% B), 20–25 min (25–35% B), 25–31 min (35–58% B), 31–34 min (58–60% B), 34–40 min (60–90% B), 40–50 min (90–10% B), and 50–60 min (10% B). 0.1% trifluoroacetic acid makes up mobile phase A and acetonitrile makes up phase B. By comparing the HPLC chromatogram with a standard curve, the result was measured and expressed as milligrams per gram of extract.

### 2.11. Statistical Analysis

All experimental results were derived from three independent replicates (*n* = 3) and are shown as mean ± standard deviation (SD). Statistical analyses were conducted using IBM SPSS Statistics (version 22.0). Prior to analysis, the assumptions for parametric testing were validated: normality was established by the Shapiro–Wilk test, and homogeneity of variances was evaluated using Levene’s test. A Two-Way Analysis of Variance (ANOVA) was utilized to assess the primary impacts of solvent type and extraction method, together with their interaction effects, on TPC and TFC. The Tukey Honestly Significant Difference (HSD) post hoc test was employed for multiple-group comparisons. Pearson correlation analysis was conducted to assess the link between extraction parameters and phytochemical yields. Student’s *t*-test was employed for comparisons between two distinct independent groups when relevant. All statistical significances were assessed at a 95% confidence level (*p* < 0.05).

## 3. Results and Discussion

### 3.1. Extraction of Plant Materials and Percentage Yield

**Figure 2:**
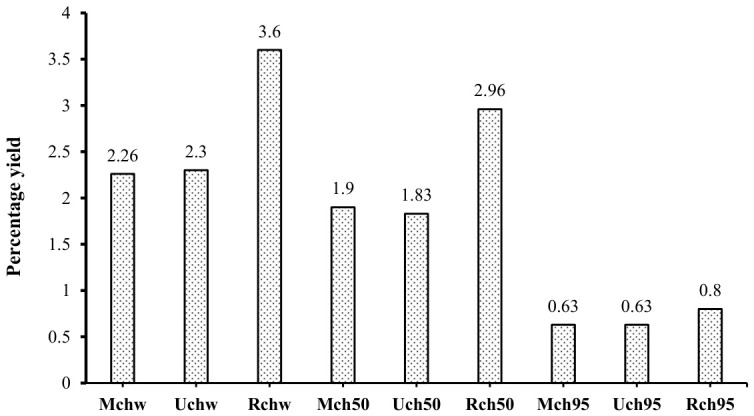
Percentage yield of corn husk extracts with different extraction methods and solvents. Mchw = maceration with water; Uchw = ultrasound-assisted extraction with water; Rchw = reflux extraction with water; Mch50 = maceration with 50% ethanol; Uch50 = ultrasound-assisted extraction with 50% ethanol; Rch50 = reflux extraction with 50% ethanol; Mch95 = maceration with 95% ethanol; Uch95 = ultrasound-assisted extraction with 95% ethanol; and Rch95 = reflux extraction with 95% ethanol. The percentage yield was calculated based on the initial dry weight of the raw material prior to extraction.

The corn husk extracts were successfully prepared under various conditions, and their yields are shown in Figure 2. Water, 50% ethanol, and 95% ethanol were selected as extraction solvents to provide different polarity systems for evaluating their effects on the extraction efficiency of bioactive compounds from corn husks. Water represents a highly polar solvent, whereas 95% ethanol represents a less polar solvent system. Meanwhile, 50% ethanol is widely used for the extraction of phenolic and flavonoid compounds because the combination of water and ethanol can improve plant matrix swelling and enhance the solubilization of compounds with intermediate polarity. Therefore, these solvent systems were chosen to investigate the influence of solvent polarity on phytochemical content and biological activities of the extracts. In the case of the reflux method, reflux extraction was conducted at 50 ± 2 °C as a mild heating condition to enhance extraction efficiency while minimizing possible thermal degradation of heat-sensitive phytochemical compounds. The percent yields for each solvent showed that maceration and ultrasound-assisted extraction yielded corn husk with similar percent yields, whereas the highest percent yield was obtained by the water reflux method. It indicated that thermal energy significantly outperformed mechanical/acoustic energy and passive diffusion in this specific aqueous setup. It is most likely that high temperatures are required to break down the lignocellulosic structure of the corn husk to release the extractives. Moreover, reflux provides constant thermal energy and solvent turnover, which maximizes the concentration gradient, while the frequency, power, or duration of ultrasound treatment might not have been sufficient to overcome the tough, fibrous nature of corn husks [5,19].

The extraction using various solvents of corn husk revealed that the corn husk extracts obtained via the water reflux method (Rchw), 50% ethanol reflux method (Rch50), ultrasound-assisted water (Uchw), and water maceration method (Mchw) yielded high percentages of 3.60%, 2.96%, 2.30%, and 2.26%, respectively. In each method, water provides the greatest percentage yield of crude corn husk extracts. The effect of solvent polarity indicated that the chemical content was extracted more effectively by polar solvents. There are reports indicating that corn husk is rich in several elements, which supply an ideal matrix for an important bioactive substance found in corn husk [14,15]. Elevated-temperature water is previously reported as the effective solvent for extracting polysaccharides [18], and the isolated polysaccharide exhibited an increase of over 30% in the weight of the fresh material when extracted at higher temperatures [20,21].

### 3.2. TPC and TFC Investigation

Table 1 demonstrates TPC in corn husk extracts. The results showed that ultrasound-assisted extraction using water (Uchw) and maceration with water (Mchw) yielded the highest TPC, at 17.30 ± 2.24 and 17.13 ± 1.60 mg GAE/g extract, respectively, with no significant difference between them. In contrast, ultrasound-assisted extraction using 95% ethanol (Uch95) resulted in the lowest TPC (2.80 ± 0.10 mg GAE/g extract). In addition, the highest TFC was found in corn husk extract obtained by ultrasound-assisted extraction by 50% ethanol (Uch50) at 126.42 ± 12.34 mg QE/g extract, followed by reflux corn husk extract with the same solvent at 113.25 ± 14.56 mg QE/g extract, whereas markedly lower values were observed in extracts obtained by ultrasound-assisted extraction with water (Uchw) and 95% ethanol (Uch95) at the values of 15.50 ± 5.88 and 17.08 ± 6.57 mg QE/g extract, respectively.

Two-way ANOVA revealed that both solvent (F = 161.54, *p* < 0.01) and extraction method (F = 15.99, *p* < 0.01) significantly affected TPC. Moreover, a significant interaction between solvent and extraction method was observed (F = 80.213, *p* < 0.001), indicating that the impact of the extraction process on TPC was dependent on the solvent used. Nevertheless, these results should be considered with caution because this significant interaction indicates that the effectiveness of extraction methods strongly depends on the specific solvent system. Similarly, the statistical analysis revealed that solvent had a significant effect on TFC (F = 117.282, *p* < 0.001), followed by the extraction method (F = 14.808, *p* < 0.001). Importantly, a significant interaction was also observed for TFC (F = 39.576, *p* < 0.001), indicating that the effectiveness of extraction methods varied depending on the solvent used. The model explained a high proportion of variance (R^2^ = 0.959), indicating that extraction efficiency is highly dependent on the synergistic effect between solvent polarity and extraction technique. The interaction effect was stronger for TPC (F = 80.213) than for TFC (F = 39.576), suggesting that phenolic compounds are more sensitive to the combined influence of solvent type and extraction methods.

Regarding the Pearson correlation from Table 2, the analysis of extraction factors and chemical constituents indicated that solvent type had a dominant linear relationship with TPC (r = 0.660, *p* < 0.01), whereas TFC recovery showed a more complex, non-linear response to the solvent-method combinations. Notably, no significant correlation was found between TPC and TFC (r = −0.173), suggesting they were extracted independently. The dominance of the solvent effect identified in the two-way ANOVA was further corroborated by this Pearson correlation. The notable interaction effects (solvent type × extraction method) analyzed in the ANOVA elucidate the weak correlation between TPC and TFC; the extraction conditions evaluated for phenolics do not correspond linearly to those for flavonoids. This divergence substantiates the application of a multivariate technique to precisely evaluate the extraction dynamics of corn husk bioactives.

The results of TPC indicated that water extraction produced the maximum TPC from corn husk, whereas the extraction of 50% and 95% ethanol led to a gradual reduction in TPC. This discovery suggests that most phenolic compounds in corn husks might be highly polar, thereby facilitating extraction with water. Phenolic compounds such as gallic acid, coumaric acid, and other hydroxycinnamic acid derivatives have previously been reported in corn waste [22]. Gallic acid contains multiple hydroxyl groups, resulting in relatively high polarity and solubility in polar solvents, whereas cinnamic acid and coumaric acid are less polar and are more readily extracted using organic solvents such as ethanol [23]. Additionally, the polarity of water can facilitate the solubilization and diffusion of polar compounds from the plant matrix [24]. Conversely, as ethanol concentration increases, solvent polarity decreases, resulting in reduced solubility of polar phenolics and, consequently, lower TPC extraction yields. Both elevated temperature and ultrasound-assisted extraction increased TPC, except for water extraction. The enhancements can be postulated to augment mass transfer and cell wall breakdown and improve diffusion of phenolic chemicals into the solvent. Ultrasound cavitation can disrupt plant cell structures and liberate bound phenolics, while heating softens plant tissue and reduces solvent viscosity, hence enhancing extraction kinetics [19]. In the case of water, elevated temperatures did not enhance TPC extraction, perhaps due to thermal breakdown or oxidation of heat-sensitive phenolics in aqueous media where solubilized oxygen can readily react with the active compounds [25]. Therefore, prolonged exposure to elevated temperatures may diminish extraction efficiency by degrading specific phenolic compounds, as water can facilitate hydrolysis and oxidation [26,27]. The superiority of 50% ethanol across all extraction methods reinforces the importance of solvent polarity in flavonoid recovery. It might be the mixture of alcohol and water that often extracts flavonoids effectively because it can dissolve both slightly polar and nonpolar compounds. A prior study indicates that an aqueous ethanol solvent can efficiently extract flavonoid chemicals from plant material. An aqueous solution containing 60% ethanol effectively extracted flavonoids in plant leaves. The yield remained constant across ethanol concentrations of 60–90% in water [28,29].

In contrast to phenolics, ultrasound-assisted extraction markedly enhanced total flavonoid content in 50% ethanol, whereas it decreased in 95% ethanol and water. The observed increase with 50% ethanol is likely due to enhanced cell rupture and improved release of moderately polar flavonoids that are effectively solubilized in mixed solvents [30]. Nonetheless, in 95% ethanol or pure water, cavitation may induce localized high temperatures and facilitate oxygen diffusion, leading to the oxidative degradation of sensitive flavonoids [31]. Consequently, increasing the extraction temperature resulted in a decline in total flavonoid content (TFC) across all solvent systems compared to ambient conditions, further confirming the thermolability of numerous flavonoid compounds [19]. Flavonoids, especially those containing numerous hydroxyl groups and conjugated double bonds, are susceptible to oxidation, polymerization, and structural deterioration at elevated temperatures [27,32]. The observed discrepancies in Uch50 might result from the balance between extraction enhancement and chemical degradation. The 50% ethanolic medium appears to provide a protective effect or a favorable equilibrium where the mechanical benefits of cavitation outweigh or offset the thermal-induced degradation of flavonoids [33].

Although flavonoids are classified as phenolic compounds, the numerical values of TFC were higher than those of TPC in some extracts. This difference may be attributed to the distinct analytical principles and calibration standards used in the assays. The TPC assay was expressed as gallic acid equivalents (GAE), whereas the TFC assay was expressed as quercetin equivalents (QE), which have different response factors and calibration characteristics. Therefore, the absolute numerical values of TPC and TFC should not be directly compared; instead, the assays are more appropriately used to evaluate relative extraction trends among different extraction conditions.

### 3.3. Antioxidant Activities

The antioxidant activity of corn husk extracts, quantified as multiples of the ascorbic acid equivalent, varied significantly with solvent type and extraction method. The corn husk extract obtained through reflux with 95% ethanol (Rch95) showed the highest free radical scavenging activity, with an IC_50_ value of 0.01 ± 0.01 mg/mL, while ascorbic acid exhibited an IC_50_ value of 0.40 ± 0.03 mg/mL as indicated in Table 3. Corn husk extracts obtained with 95% ethanol had antioxidant activity that was 6 to 40 times more effective than ascorbic acid, whereas extracts obtained with 50% ethanol showed activity that was 3 to 20 times greater. Reflux extraction with 95% ethanol (Rch95) produced the maximum antioxidant activity, measuring 40 times that of ascorbic acid, followed by ultrasound-assisted extraction with 50% ethanol (Uch50) and water maceration (Mchw). Conversely, ultrasound-assisted water (Uchw) extraction exhibited the lowest activity (0.77-fold). The variations between DPPH assay and TPC/TFC values might arise from the nature of the chemical compounds and the specifics of the analytical assays. Although water effectively extracts phenolic compounds from corn husk, the water extract may not necessarily exhibit the highest antioxidant activity. This is because the Folin–Ciocalteu assay measures the total reducing capacity of compounds containing hydroxyl groups, which does not always directly correlate with radical scavenging potency. Differences in antioxidant activity among extracts may instead be associated with variations in the composition, structure, and reactivity of individual phenolic and flavonoid compounds extracted under different solvent conditions. Therefore, ethanolic extracts may exhibit higher DPPH radical scavenging activity despite showing lower total phenolic content. The impact of extracts on lipid degradation was evaluated by the ferric thiocyanate assay [34]. This study revealed that the high potency of lipid peroxidation inhibition was from ascorbic acid and corn husk extract from maceration (Mch95) and reflux (Rch95) with 95% ethanol at IC_50_ values of 0.08 ± 0.06 and 0.01 ± 0.01 mg/mL, respectively.

The most significant reduction in lipid peroxidation was observed with 95% ethanol maceration (Mch95) and reflux extraction (Rch95), with each of them showing an 8-fold increase in activity compared to ascorbic acid. The findings indicate that lipophilic flavonoids extracted with 95% ethanol are more efficacious at the lipid–aqueous interface [35], where they can inhibit the peroxidation of lipid substrates. In contrast, the 50% ethanol extract exhibited minimal to no inhibition (0.38-fold relative to the control), likely because the hydro-ethanolic solvent system was less effective at extracting the specific phenolic acids required to inhibit lipid peroxidation. This lack of activity may stem from a lower concentration of key bioactive compounds compared to the other solvent systems.

### 3.4. Anti-Tyrosinase Activity

There are reports indicating that antioxidant activity is usually positively correlated with anti-tyrosinase activity [34,35], particularly through lipid peroxidation, which indirectly affects tyrosinase activity [36]. Hence, the antioxidant activity of the corn husk extract, obtained by maceration and reflux with 50% ethanol, was excluded from further studies due to its ineffectiveness in lipid peroxidation. Tyrosinase is a multifunctional enzyme responsible for melanin biosynthesis, exhibiting both monophenolase and diphenolase activities. In melanogenesis, the monophenolase activity, which converts L-tyrosine to L-DOPA, represents the rate-limiting step, whereas diphenolase activity catalyzes the following oxidation of L-DOPA to dopaquinone. These two catalytic functions differ in their kinetic characteristics and inhibitor sensitivity, enabling selective inhibition of specific stages of melanin formation. The tyrosinase inhibition of various compounds achieved by using both substrates clarifies their efficacy and selectivity toward various enzymatic activities [37]. As a result, the discovery of a tyrosinase inhibitor has emerged as a critical area of research for reducing skin pigmentation [38]. Table 4 presents the IC50 values of ascorbic acid and arbutin, as measured by L-tyrosine and L-DOPA. The ultrasound-assisted water extract (Uchw) exhibited an IC_50_ value of 7.54 ± 0.27 mg/mL for L-DOPA and 0.10 ± 0.02 mg/mL for L-tyrosine. The selectivity index analysis revealed a distinct inhibitory profile among the tested samples. Uchw exhibited an exceptionally low selectivity index at 0.013, indicating a strong preference for inhibiting monophenolase activity over diphenolase activity. This suggests that the extract is more effective at suppressing the initial step of melanogenesis, where L-tyrosine is converted to L-DOPA. Such selective inhibition is particularly valuable, as targeting monophenolase activity can prevent the onset of melanin biosynthesis rather than merely slowing downstream oxidation processes. In contrast, ascorbic acid displayed a selectivity index close to unity at 1.05, indicating non-selective inhibition toward both substrates. This behavior is consistent with its well-known role as a reducing agent that interferes with multiple stages of the tyrosinase catalytic cycle. Arbutin, on the other hand, showed weak activity under the tested conditions, particularly against L-DOPA, thereby preventing reliable assessment of selectivity. Overall, these findings highlight Uchw as a promising candidate with selective monophenolase inhibitory activity, which may offer advantages in cosmetic or dermatological applications aimed at controlling hyperpigmentation. In addition, the results indicate that the tyrosinase-inhibitory chemicals in corn husk are likely polar phenolic compounds, which are effectively extracted with ultrasound treatment in water.

These phenolic compounds may function as competitive or chelating inhibitors, inhibiting melanin synthesis [39]. A study showed that water-soluble corn silk extract significantly reduced melanin production in Melan-A cells. Corn silk is believed to impede melanin synthesis by downregulating tyrosinase expression at the cellular level [40]. Meanwhile, corncob extract also presented anti-tyrosinase activity correlating with flavonoid and phenolic contents [12]. This observation contrasts notably with the antioxidant findings, in which refluxed 95% ethanol extracts exhibited superior efficacy. This distinction emphasizes that tyrosinase inhibition does not directly correlate with total antioxidant capacity or flavonoid content, but it is contingent on the presence of specific polar phenolic inhibitors [41,42].

### 3.5. NO Scavenging Activity

**Figure 3:**
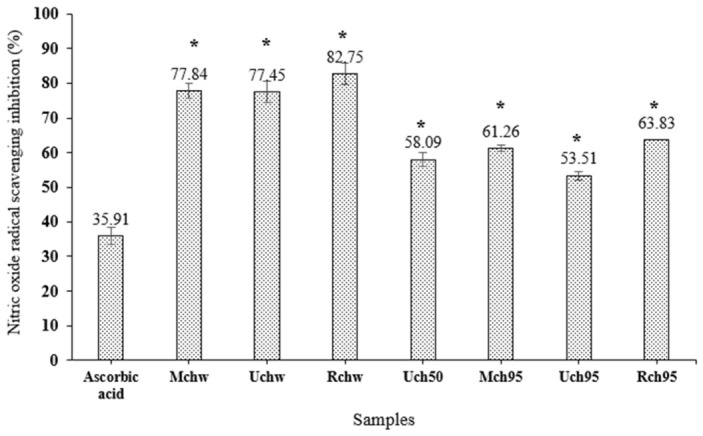
Nitric oxide radical scavenging activity of corn husk extracts and ascorbic acid. All extracts and ascorbic acid were evaluated at a concentration of 10 mg/mL. * Significantly different from ascorbic acid at *p* < 0.05 (Student’s *t*-test). Mchw = maceration with water; Uchw = ultrasound-assisted extraction with water; Rchw = reflux extraction with water; Mch50 = maceration with 50% ethanol; Uch50 = ultrasound-assisted extraction with 50% ethanol; Rch50 = reflux extraction with 50% ethanol; Mch95 = maceration with 95% ethanol; Uch95 = ultrasound-assisted extraction with 95% ethanol; and Rch95 = reflux extraction with 95% ethanol.

NO is an essential chemical involved in anticancer properties, blood pressure control, and facilitation of brain signal transduction. Minimal concentrations of NO are frequently sufficient to elicit these advantageous effects [43]. In contrast, nitric oxide production is significantly elevated during infections and inflammations, potentially leading to adverse effects [44]. The capacity of plants or plant derivatives to mitigate nitric oxide synthesis may be significant in reducing the harmful effects of excessive nitric oxide that occur in the body. Furthermore, the scavenging activity may assist in averting the cascade of adverse health responses induced by the overproduction of NO [43]. NO inhibition results indicated that corn husk extracts derived from water-based extraction methods, including maceration, reflux, and ultrasound-assisted techniques, displayed superior inhibitory effectiveness, surpassing that of ascorbic acid, as illustrated in Figure 3.

This study indicates that the primary chemicals responsible for NO inhibition are highly polar phenolics, which are effectively removed in aqueous solutions. Phenolic acids, including ferulic acid and p-coumaric acid, are prevalent in corn husk, are recognized for their anti-inflammatory and free radical-scavenging characteristics [45]. These chemicals can regulate nitric oxide synthesis by suppressing the expression of inducible nitric oxide synthase (iNOS) or by directly neutralizing reactive nitrogen species, resulting in reduced NO levels [43]. The elevated NO inhibition noted in water extracts, in comparison to ethanol extracts, aligns more closely with the trend of TPC than with TFC. The water-extracted fraction, which produced the highest TPC, indicates that phenolic acids play a more substantial role in nitric oxide (NO) inhibition than less-polar flavonoids.

For the bioactivity screening of antioxidant, nitric oxide inhibitory, and anti-tyrosinase activities, the ultrasound-assisted water extraction (Uchw) approach was selected for further study of corn husk extract due to its effective release of phenolic compounds using a safe, environmentally friendly solvent. This approach yielded extracts with significant inhibition of nitric oxide and tyrosinase. Despite having lower antioxidant potential than ethanol extracts, the technology provides a sustainable and efficient method for obtaining bioactive corn husk extracts suitable for health applications.

### 3.6. Anti-Aging Activities

The concentration of ultrasound-assisted water extract (Uchw) used for the collagenase and hyaluronidase inhibition assays was 5 mg/mL. The inhibitory activities are presented in Table 5. In the collagenase inhibition assay, ascorbic acid (2 mg/mL) and EGCG (1 mg/mL) exhibited inhibition values of 29.79 ± 1.00% and 82.27 ± 1.00%, respectively. Under the tested conditions, Uchw demonstrated hyaluronidase inhibition of 2.88 ± 1.99%, whereas ascorbic acid and EGCG showed inhibition values of 0.88 ± 0.53% and 1.41 ± 1.34%, respectively. In addition, Uchw exhibited collagenase inhibition of 91.49 ± 4.01%, compared with 29.79 ± 1.00% for ascorbic acid and 82.27 ± 1.00% for EGCG.

However, these results should be interpreted cautiously because the extract and reference compounds were tested at different concentrations; therefore, direct comparison of inhibitory potency may be limited. Further dose–response studies and IC_50_ determination are necessary for more rigorous comparison of anti-aging activity. Collagenase is an important enzyme that is involved in skin aging through degradation of collagen in the extracellular matrix. Hyaluronidase degrades hyaluronic acid, which plays a key role in maintaining skin hydration and structural integrity. Therefore, inhibition of these enzymes may contribute to anti-aging effects and reduction in age-related skin deterioration [14,46].

### 3.7. HET-CAM Study

Irritation is a crucial factor in the formulation of topical therapies. This experiment demonstrates an adverse effect on the CAM of viable eggs treated with a substance [47]. The irritating properties of corn husk extract were assessed using the HET-CAM assay. The HET-CAM assay was adopted as a non-animal surrogate for the Draize test to assess the irritancy of cosmetic formulations [48]. Its application underscores the importance of rigorous pre-clinical safety screening before proceeding with clinical investigations [49]. The corn husk extract obtained via ultrasound-assisted aqueous extraction was selected for further analysis. Table 6 shows no irritation in the negative control. The well-known irritant, 1% *w*/*w* sodium lauryl sulfate (SLS), caused substantial irritation with an irritation score (IS) of 19.7 ± 0.8.

**Figure 4:**
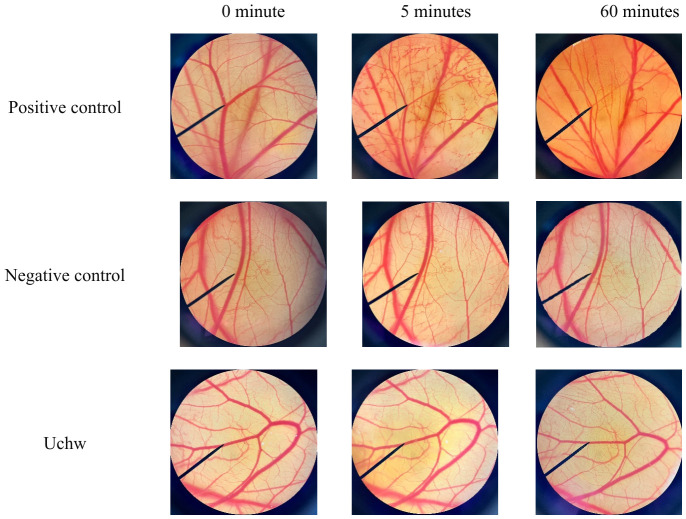
HET-CAM response to positive control (1% *w*/*v* sodium lauryl sulfate) and negative control (0.9% *w*/*v* normal saline solution) at various times of exposure; Uchw = ultrasound-assisted extraction with water.

In contrast, the negative control (0.9% *w*/*w* normal saline solution) exhibited no signs of irritation, yielding a score of 0.0 ± 0.0. Conversely, the SLS solution induced pathological changes in the chorioallantoic membrane damage, including rapid hemorrhage, subsequently escalating to coagulation and lysis of the vessels. These irritant-induced markers were detectable within five minutes and became significantly more pronounced after sixty minutes, as illustrated in Figure 4. A concentration of 10 mg/mL of the corn husk extract, obtained by ultrasound-assisted water extraction, was evaluated for its capacity to induce irritation. The CAM treated with corn husk extract showed no indications of irritation following five and sixty minutes of treatment. As a result, Uchw extracts did not provoke any irritation and were considered safe for continued use.

### 3.8. Cell Viability Assessment

**Figure 5:**
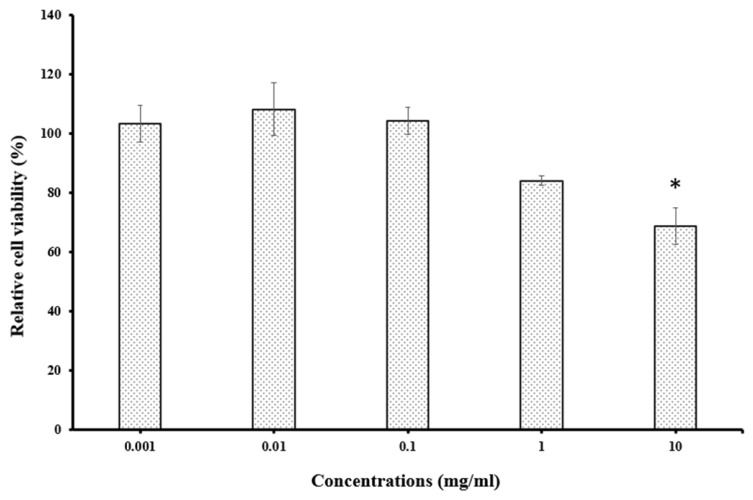
The percentage of human dermal fibroblast cell viability when treated by corn husk extract at various concentrations; * = substantially below 80% when compared to the control cells.

In addition to the HET-CAM assay, the safety of corn husk extract performed by ultrasound-assisted methods with water was evaluated using a cytotoxicity test. Cellular safety was characterized through an MTT-based viability assay on human dermal fibroblasts, with non-cytotoxic levels defined as those maintaining at least 80% viability relative to the control [17]. This conventional approach exploits the metabolic capability of living cells to convert yellow tetrazolium salt into purple formazan. Thus, the amount of formazan production was assessed as a direct measure of the viable cells and overall cellular viability [17]. As illustrated in Figure 5, the Uchw extract at 10 mg/mL exhibited cytotoxicity to the cell lines, with cell viability obviously below 80%, following a 24 h incubation with the samples on the cells.

The study demonstrated that Uchw at 10 mg/mL exhibited cytotoxicity in cell-based assays, whereas the HET-CAM test showed no irritation at the same concentration. This suggests that although the extract may affect cellular viability under in vitro conditions, possibly due to high levels of phenolic compounds or reactive metabolites, it does not cause acute irritation or damage to biological membranes. The discrepancy may result from differences in exposure conditions and biological models: the cytotoxicity test reflects cellular metabolic response [17,50], whereas HET-CAM assesses tissue irritation [47]. Therefore, the extract appears non-irritating but may require concentration optimization for safe biological applications.

### 3.9. Analysis of Active Content by the HPLC Assay

**Figure 6:**
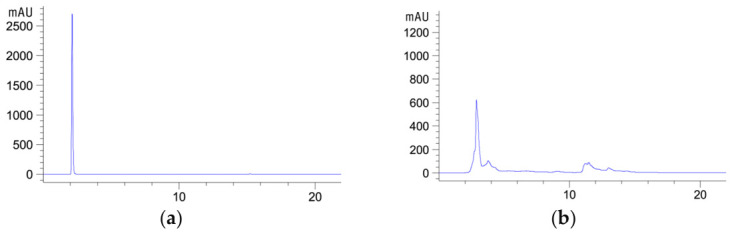
Chromatogram of gallic acid in 0.5 mg/mL standard solution (**a**) and 10 mg/mL Uchw (**b**).

**Figure 7:**
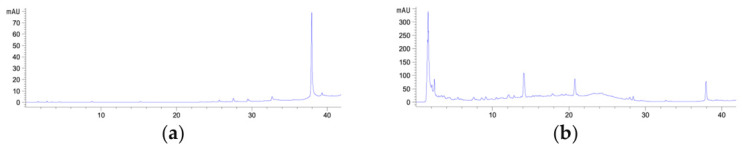
Chromatogram of quercetin in 2.0 mg/mL standard solution (**a**) and 10 mg/mL Uchw (**b**).

Numerous bioactive characteristics had been established by the phytochemical investigation of corn husk extracts. Corn husk may be preferred for pharmaceutical purposes according to the presence of valuable phytochemical substances [45,51]. Gallic acid and quercetin in Uchw were successfully determined by HPLC. The chromatograms of gallic acid and quercetin are presented in Figure 6 and Figure 7, respectively.

The amounts of active contents in the corn husk extract obtained by ultrasound-assisted water extraction (Uchw) are shown in the HPLC results as presented in Table 7.

Uchw possesses a gallic acid concentration of 17.21 ± 0.34 mg/g extract. Furthermore, one of the main naturally occurring flavonols, quercetin, is categorized as a member of the flavonoid family [52]. It has historically been a part of typical human nutrition [53]. Uchw also contains 63.09 ± 19.12 mg/g of quercetin. It’s interesting to note that the Uchw extract showed apparent discrepancies between HPLC quantification and spectrophotometric tests. An HPLC study showed a high concentration of quercetin but a relatively low concentration of gallic acid, despite Uchw’s relatively high total phenolic content (TPC) and relatively low total flavonoid content (TFC). These variations could be explained by the different analytical principles of the techniques used. The Folin–Ciocalteu test, which is employed for TPC, is not specific to any one phenolic compound; rather, it assesses the total reducing capacity of compounds containing hydroxyl groups. Similarly, rather than reflecting the exact concentration of particular flavonoids, the aluminum chloride colorimetric assay employed for TFC represents total flavonoid reactivity. HPLC, on the other hand, measures specific molecules with greater sensitivity and specificity. Therefore, depending on the composition, structural variety, and test response variables of the phytochemical elements in the extract, the spectrophotometric assays and chromatographic analysis may yield varied quantitative patterns. Furthermore, even though the TFC assay showed very low total flavonoid reactivity, quercetin might be a major flavonoid ingredient in the Uchw extract. However, the result of the current study is also similar to previous research that suggested that the main phenolics in corn waste were quercetin and gallic acid [21,30]. Gallic acid (GA), a principal component of the phenolic group, functions as a prevalent secondary metabolite in numerous plant sources. This low-molecular-weight triphenolic compound is distinguished by its significant antioxidant and anti-inflammatory activity, differentiating it from other polyphenols [54]. Consequently, processed corn husk, which is rich in gallic acid and quercetin, may represent a valuable natural resource with interesting applications.

## 4. Conclusions

This study demonstrated that corn husk, an agricultural by-product, is a valuable source of bioactive compounds with potential antioxidant, anti-inflammatory, and skin-protective properties. Solvents and extraction techniques significantly influenced the yield and biological activities of the extracts. Water extraction provided the highest total phenolic content, whereas ethanol extraction, particularly with 95% ethanol, was more effective for flavonoid recovery and antioxidant potential. The 95% ethanol extract exhibited the strongest DPPH radical scavenging and lipid peroxidation inhibition, while water extracts, especially those obtained by ultrasound-assisted water extraction (Uchw), showed superior nitric oxide and tyrosinase inhibition activities. The ultrasound-assisted water extraction method effectively enhanced the release of gallic acid and quercetin while maintaining mild, solvent-free conditions. This extract demonstrated notable biological activities, including strong inhibition of nitric oxide production and potent anti-collagenase effects, suggesting potential anti-aging benefits through collagen protection. The HET-CAM assay demonstrated non-irritating properties at 10 mg/mL; however, the cytotoxicity observed at this same concentration indicates a threshold where cellular viability is compromised. Consequently, although the extract shows promise for topical application, the precise safety-to-efficacy margin and optimal dose–response relationship require further investigation through more complex dermatological models. Therefore, Uchw is recommended as an eco-friendly, safe, and effective method for producing corn husk extracts with multifunctional bioactivities, including antioxidant, anti-inflammatory, and anti-aging effects, making it suitable for potential use in functional food and cosmetic formulations.

## 5. Limitation

This study primarily focused on phenolic and flavonoid compounds through TPC and TFC determination. However, polysaccharides may also be present, particularly in aqueous extracts, and could contribute to the observed biological activities. Nevertheless, polysaccharide characterization was beyond the scope of the present study and should be investigated in future research. In addition, because the extracts and reference standards were evaluated at different concentrations, the inhibition percentages should be interpreted as preliminary comparative data rather than direct potency equivalence. Therefore, further dose–response studies and IC_50_ determination are required for more accurate comparison of inhibitory activities. Moreover, the HPLC analysis focused only on gallic acid and quercetin; therefore, the complete phytochemical profile of the extracts was not fully characterized. Further comprehensive chromatographic analysis is needed to identify additional bioactive compounds.

## Figures and Tables

**Table 1:** TPC and TFC of corn husk extract determined using various extraction methods.

Solvent	Total Phenolic Content (mg GAE/g Extract)			Total Flavonoid Content (mg QE/g Extract)		
	Maceration	Ultrasound-Assisted	Reflux	Maceration	Ultrasound-Assisted	Reflux
95% ethanol	3.16 ± 0.55 ^c^	2.80 ± 0.10 ^c^	7.22 ± 0.64 ^b^	71.58 ± 4.29 ^d^	17.08 ± 6.57 ^e^	44.08 ± 6.66 ^g^
50% ethanol	5.88 ± 0.08 ^b^	6.49 ± 1.08 ^b^	7.76 ± 0.22 ^b^	73.00 ± 6.31 ^d^	126.42 ± 12.34 ^f^	113.25 ± 14.56 ^f^
Water	17.13 ± 1.60 ^a^	17.30 ± 2.24 ^a^	4.23 ± 0.38 ^c^	82.92 ± 11.55 ^d^	15.50 ± 5.88 ^e^	51.17 ± 9.06 ^g^

Note: The data was shown as mean ± SD (*n* = 3). Statistical significance at *p* < 0.05 among groups in the same column was denoted by superscript letters with comparisons evaluated by Tukey’s HSD test in two-way ANOVA.

**Table 2:** Pearson correlation coefficients among TPC, TFC, extraction method and solvent type (*n* = 27).

Variables	TPC	TFC	Extraction Method	Solvent Type
TPC	1.000	−0.173	−0.180	0.660 *
TFC	−0.173	1.000	−0.070	−0.073
Extraction method	−0.180	−0.070	1.000	0.000
Solvent type	0.660 *	−0.073	0.000	1.000

* Correlation is significant at the 0.01 level (2-tailed).

**Table 3:** The IC_50_ values for antioxidant activity.

Samples	DPPH (mg/mL)	Ascorbic Acid-Equivalent Potency (Fold)	Lipid Peroxidation Inhibition (mg/mL)	Ascorbic Acid-Equivalent Potency (Fold)
Mchw	0.03 ± 0.01	13.33	0.02 ± 0.01	4.00
Uchw	0.52 ± 0.20	0.77	0.03 ± 0.01	2.67
Rchw	ND	-	0.03 ± 0.02	2.67
Mch50	0.11 ± 0.01	3.64	ND	-
Uch50	0.02 ± 0.01	20.00	0.21 ± 0.10	0.38
Rch50	0.05 ± 0.01	8.00	ND	-
Mch95	0.06 ± 0.03	6.67	0.01 ± 0.01	8.00
Uch95	0.05 ± 0.01	8.00	0.08 ± 0.02	1.00
Rch95	0.01 ± 0.01	40.00	0.01 ± 0.01	8.00
Ascorbic acid	0.40 ± 0.03	-	0.08 ± 0.06	-

Note: ND = not detected. Mchw = maceration with water; Uchw = ultrasound-assisted extraction with water; Rchw = reflux extraction with water; Mch50 = maceration with 50% ethanol; Uch50 = ultrasound-assisted extraction with 50% ethanol; Rch50 = reflux extraction with 50% ethanol; Mch95 = maceration with 95% ethanol; Uch95 = ultrasound-assisted extraction with 95% ethanol; and Rch95 = reflux extraction with 95% ethanol. Ascorbic acid equivalent potency (fold) = $\frac{IC50\;of\;ascorbic\;acid}{IC50\;of\;corn\;husk\;extract}$.

**Table 4:** The IC_50_ values for anti-tyrosinase activity.

Samples	IC_50_ (mg/mL)		Selectivity Index (L-Tyrosine/L-Dopa)	Interpretation
	L-DOPA	L-Tyrosine		
Mchw	>1000	>1000	-	Inactive
Uchw	7.54 ± 0.27	0.10 ± 0.02	0.013	Strong monophenolase selective
Rchw	ND	>1000	-	Inactive
Uch50	>1000	ND	-	Inactive
Mch95	ND	>1000	-	Inactive
Uch95	>1000	>1000	-	Inactive
Rch95	>1000	>1000	-	Inactive
Ascorbic acid	0.20 ± 0.05	0.21 ± 0.09	1.05	Non-selective
Arbutin	>1000	1.13 ± 0.24	-	Inactive

Note: ND = not detected. Mchw = maceration with water; Uchw = ultrasound-assisted extraction with water; Rchw = reflux extraction with water; Mch50 = maceration with 50% ethanol; Uch50 = ultrasound-assisted extraction with 50% ethanol; Rch50 = reflux extraction with 50% ethanol; Mch95 = maceration with 95% ethanol; Uch95 = ultrasound-assisted extraction with 95% ethanol; and Rch95 = reflux extraction with 95% ethanol.

**Table 5:** Anti-aging effects of corn husk extracts on hyaluronidase and collagenase.

Samples	% Hyaluronidase Inhibition	% Collagenase Inhibition
Uchw (5 mg/mL)	2.88 ± 1.99	91.49 ± 4.01 *^,^**
Ascorbic acid (2 mg/mL)	0.88 ± 0.53	29.79 ± 1.00
EGCG (1 mg/mL)	1.41 ± 1.34	82.27 ± 1.00

Note: * and ** indicated significant difference at *p* < 0.05 compared to ascorbic acid and EGCG, respectively; Uchw = ultrasound-assisted extraction with water.

**Table 6:** Irritation severity score of corn husk extract (Uchw), sodium lauryl sulfate, and normal saline according to HET-CAM assay.

Samples	Irritation Score
Sodium lauryl sulfate (1% *w*/*w*)	19.7 ± 0.8
Normal saline solution (0.9% *w*/*w*)	0.0 ± 0.0
Uchw (10 mg/mL)	0.0 ± 0.0

Uchw = ultrasound-assisted extraction with water.

**Table 7:** Quantity of active contents in corn husk extracts as determined by HPLC analysis.

Sample	Gallic Acid Concentration (mg/g Extract ± SD)	Quercetin Concentration (mg/g Extract ± SD)
Uchw	17.21 ± 0.34	63.09 ± 19.12

Uchw = ultrasound-assisted extraction with water.

## Data Availability

The original contributions presented in this study are included in the article. Further inquiries can be directed to the corresponding author.
